# Risk Factors and Predictive Accuracy of the Rotterdam Risk Index for Wound Dehiscence Following Abdominal Surgery

**DOI:** 10.7759/cureus.76769

**Published:** 2025-01-01

**Authors:** Rajat Sharma, Siddharth B Lonare, Pratul Arora, Hamza Al-Dwlai, Alpa Vadher, Mohamed Hersi

**Affiliations:** 1 General Surgery, Luton and Dunstable University Hospital, Luton, GBR; 2 General Surgery, Maharishi Markandeshwar Medical College and Hospital, Solan, IND; 3 General Surgery, Byramjee Jeejeebhoy Government Medical College and Sassoon General Hospital, Pune, IND; 4 General Surgery, Fortis Hospital, Ludhiana, IND; 5 General Internal Medicine, Luton and Dunstable University Hospital, Luton, GBR

**Keywords:** postoperative complications, risk factors, rotterdam risk index, serum albumin, wound dehiscence

## Abstract

Background

Wound dehiscence (WD) is a major postoperative complication following abdominal surgeries, particularly exploratory laparotomy. Identifying preoperative risk factors and using predictive tools, such as the Rotterdam Risk Index (RRI), are crucial for early intervention and improving patient outcomes. This study aimed to evaluate the risk factors associated with WD and assess the predictive accuracy of the RRI in a cohort of patients undergoing abdominal surgeries.

Methods

This retrospective observational study included 151 patients who underwent exploratory laparotomy at a tertiary care hospital. Demographic details, comorbidities, surgical factors, and postoperative complications were recorded. The RRI was calculated preoperatively for each patient. WD was diagnosed based on clinical signs and confirmed through physical examination. Statistical analysis was performed using SPSS software to determine the associations between various risk factors and the occurrence of WD.

Results

The study identified several factors significantly associated with WD, including male gender, emergency surgery, low serum albumin levels (<3.5 g/dL), anemia (hemoglobin <10 g/dL), and wound contamination. Male patients had a higher risk of WD, with odds of 1.9 (95% confidence interval (CI): 1.1-3.3, p = 0.021). Emergency surgery was associated with a higher incidence of WD (odds ratio (OR): 4.1, 95% CI: 1.5-10.4, p = 0.017). The RRI showed high sensitivity (100%) and specificity (90.2%) for predicting WD preoperatively, with an area under the ROC curve of 0.986. Postoperatively, 22 patients with WD were treated with resuturing, while two required reoperation due to anastomotic leaks.

Conclusion

The RRI demonstrated excellent predictive accuracy for identifying patients at high risk of WD before surgery. Early identification of risk factors, such as low serum albumin, anemia, and emergency surgeries, enables personalized perioperative management strategies, including nutritional optimization and careful intraoperative monitoring, which can significantly reduce the risk of WD. These findings emphasize the clinical utility of the RRI in guiding surgical decision-making and improving patient outcomes.

## Introduction

Abdominal wound dehiscence (AWD) is a grave postoperative complication, defined as the partial or complete disruption of fascial layers after abdominal surgery, often occurring within the first two weeks of operation. It is associated with significant morbidity, mortality rates ranging between 10% and 40%, prolonged hospital stays, and increased healthcare costs [1,2]. The reported global incidence of AWD ranges from 0.2% to 5%, with studies from India reporting a higher prevalence of approximately 5-10% in emergency laparotomies, largely attributed to suboptimal nutritional status, infection control challenges, and limited access to healthcare resources [3,4].

Numerous risk factors contribute to AWD, broadly categorized into patient-related, surgery-related, and postoperative factors. Patient-related factors include advanced age, male sex, hypoalbuminemia, anemia, diabetes mellitus, and smoking. For instance, hypoalbuminemia (<3.5 g/dL) has been reported as a strong predictor, with odds ratios (OR) ranging from 2.5 to 6.0 in various studies [5,6]. Surgery-related factors such as emergency procedures, contaminated wounds, prolonged operative time (>2 hours), and improper closure techniques significantly elevate the risk. Among these, emergency laparotomy carries a 10-fold higher risk compared to elective procedures [7]. Postoperative complications like surgical site infections (SSIs) further aggravate the risk, with studies showing an AWD incidence of 15-20% in cases complicated by SSIs [8].

The Rotterdam Risk Index (RRI) model, developed in 2013, has emerged as a reliable tool for predicting the risk of AWD. The RRI integrates clinical and perioperative variables such as age, body mass index (BMI), anemia, hypoalbuminemia, presence of SSI, and surgical wound classification into a composite score [9]. A validation study demonstrated that patients with an RRI score above 10 had a 40% risk of AWD, compared to 5% in those with lower scores [10]. Despite its potential, its applicability in resource-constrained settings like India remains underexplored.

This study aimed to evaluate the predictive accuracy of the RRI model in identifying risk factors associated with AWD in an Indian cohort. By doing so, it seeks to identify modifiable risk factors and assess the feasibility of using this model to guide preoperative and postoperative interventions in resource-limited environments.

## Materials and methods

Study design and setting

This was a retrospective observational study conducted in the Department of General Surgery at Byramjee Jeejeebhoy Government Medical College and Sassoon General Hospital, Pune, over a two-year period from June 2022 to May 2024. The study was designed to assess the utility of the RRI model in predicting AWD and identifying associated risk factors. Ethical approval for the study was obtained from the Institutional Ethics Committee (approval number: BJGMCDGH/2022/1/187), and all participants provided written informed consent before enrollment.

Study population

The study included adult patients aged 18 years and older who underwent midline laparotomy for various indications during the study period. Inclusion criteria encompassed patients undergoing both elective and emergency laparotomies. Exclusion criteria included patients with pre-existing AWD, those undergoing relaparotomy for non-related causes, and patients lost to follow-up within 30 days of surgery. A total of 151 participants meeting the eligibility criteria were enrolled in the study.

Sample size calculation

The sample size was calculated using the formula for proportions, based on an expected prevalence of AWD of 5%, with a confidence interval of 95% and a margin of error of 5% [11]. This yielded a minimum sample size requirement of 130 participants. To account for potential dropouts, an additional 10% was added, resulting in a final target enrollment of 151 patients.

Data collection

Data were collected prospectively using a standardized proforma designed specifically for the study. Demographic details, including age, sex, and body mass index (BMI), were recorded at the time of enrollment. Preoperative clinical data included hemoglobin levels, serum albumin levels, and the presence of comorbidities such as diabetes mellitus, chronic obstructive pulmonary disease (COPD), and smoking history. Intraoperative details, such as the nature of surgery (elective or emergency), the duration of the procedure, the classification of the surgical wound (clean, clean-contaminated, contaminated, or dirty), and the use of drains, were meticulously documented by the surgical team. Postoperative variables, including the occurrence of SSI, mechanical ventilation, and the postoperative day of dehiscence (if applicable), were recorded during hospitalization and subsequent follow-up visits.

Risk assessment using the RRI

The RRI model was applied to each participant to assess the risk of AWD. This model uses a scoring system based on specific clinical and perioperative factors, including age (≥65 years), BMI (≥30 kg/m²), anemia (hemoglobin <10 g/dL), hypoalbuminemia (serum albumin <3.5 g/dL), emergency surgery, and the occurrence of SSI. Each parameter was assigned a predefined weight in the RRI model, and the cumulative score was calculated for all participants. Patients were categorized into two risk groups: low risk (<10 points) and high risk (≥10 points).

Follow-up

Participants were closely monitored during their hospital stay for the development of AWD or related complications. Post-discharge follow-up was conducted at regular intervals of up to 30 days through outpatient visits or telephonic consultations. Any cases of suspected AWD were re-evaluated clinically or through imaging as necessary.

Outcome measures

The primary outcome of the study was the incidence of AWD, defined as the partial or complete disruption of fascial layers confirmed clinically by wound inspection or radiologically within 30 days post-surgery. Secondary outcomes included the identification of independent risk factors associated with AWD and the evaluation of the predictive accuracy of the RRI model.

Statistical analysis

Statistical analysis was performed using IBM SPSS Statistics for Windows, version 20.0 (released 2011, IBM Corp., Armonk, NY). Continuous variables were expressed as means and standard deviations, while categorical variables were expressed as frequencies and percentages. Comparisons between groups (AWD vs. non-AWD) were made using the independent T-test for continuous variables and the chi-square test for categorical variables. Logistic regression analysis was conducted to identify independent predictors of AWD, with ORs and 95% CIs reported for each significant variable. The predictive accuracy of the RRI model was assessed using the receiver operating characteristic (ROC) curve, and the area under the curve (AUC) was calculated. A p-value of <0.05 was considered statistically significant for all analyses.

## Results

The AWD group had a mean age of 38.5 ± 10.2 years, which was slightly lower than the non-AWD group's mean age of 43.7 ± 12.4 years (p = 0.078). In terms of age distribution, 54.8% of the AWD group and 45.2% of the non-AWD group were under 40 years (p = 0.152). Gender distribution was similar between the groups, with 66.1% of the AWD group and 60.9% of the non-AWD group being male (p = 0.464). Malnutrition was significantly more common in the AWD group (37.3% vs. 26.1%, p = 0.045), while obesity rates were comparable (15.3% vs. 18.5%, p = 0.612). Tuberculosis was more prevalent in the AWD group (22.0% vs. 7.6%, p = 0.011), while other comorbidities, such as malignancy, diabetes, hypertension, and chronic kidney disease, showed no significant differences. Rates of smoking and alcohol use were also similar between the two groups. Steroid use and chemotherapy history were comparable between the AWD and non-AWD groups, with no significant differences (p = 0.231 and p = 0.346, respectively) (Table 1).

**Table 1:** Demographic and clinical characteristics of the participants. AWD: abdominal wound dehiscence; non-AWD: non-abdominal wound dehiscence

Characteristics	AWD group (n = 59)	Non-AWD group (n = 92)	Test statistics	p-value
	Frequency (%)/mean ± SD			
Age (years)	38.5 ± 10.2	43.7 ± 12.4	t = -1.770	0.078
Age group				
<40 years	32 (54.8%)	41 (45.2%)	χ² = 2.073	0.152
>40 years	27 (45.2%)	51 (54.8%)		
Gender				
Male	39 (66.1%)	56 (60.9%)	χ² = 0.518	0.464
Female	20 (33.9%)	36 (39.1%)		
Body mass index (BMI)				
Normal	28 (47.5%)	51 (55.4%)	χ² = 0.965	0.328
Malnourished	22 (37.3%)	24 (26.1%)	χ² = 4.040	0.045
Obese	9 (15.3%)	17 (18.5%)	χ² = 0.233	0.612
Smoking (current/former)	21 (35.6%)	27 (29.3%)	χ² = 0.635	0.423
Alcohol use	24 (40.7%)	32 (34.8%)	χ² = 0.759	0.389
Comorbidities				
Jaundice	4 (6.8%)	3 (3.3%)	χ² = 0.001	0.95
Tuberculosis	13 (22.0%)	7 (7.6%)	χ² = 6.376	0.011
Malignancy	7 (11.9%)	3 (3.3%)	χ² = 3.017	0.083
Diabetes mellitus	17 (28.8%)	18 (19.6%)	χ² = 2.730	0.097
Hypertension	11 (18.6%)	13 (14.1%)	χ² = 1.091	0.297
Chronic kidney disease	3 (5.1%)	2 (2.2%)	χ² = 0.319	0.576
Chronic obstructive pulmonary disease (COPD)	3 (5.1%)	2 (2.2%)	χ² = 0.319	0.576
History of previous surgery	3 (5.1%)	3 (3.3%)	χ² = 0.041	0.834
Immunosuppression				
Steroid use	7 (11.9%)	6 (6.5%)	χ² = 1.432	0.231
Chemotherapy	5 (8.5%)	5 (5.4%)	χ² = 0.881	0.346

In the study, the AWD group exhibited higher proportions of risk factors compared to the non-AWD group. Hemoglobin levels <10 g/dL were found in 54.2% of the AWD group and 34.8% of the non-AWD group (p = 0.044). Similarly, serum albumin levels <3.5 g/dL were observed in 64.4% of the AWD group, compared to 38.0% in the non-AWD group (p = 0.032). White blood cell counts >11×10⁹/L were significantly higher in the AWD group (44.1% vs. 23.9%, p = 0.015), as were ASA scores ≥3 (30.5% vs. 13.0%, p = 0.021). The incidence of anemia was also higher in the AWD group (61.0% vs. 35.9%, p = 0.011). Preoperative infection was more prevalent in the AWD group (69.5% vs. 32.6%, p < 0.001), and nutritional supplementation before surgery was more common in the AWD group (32.2% vs. 16.3%, p = 0.045) (Table 2).

**Table 2:** Preoperative risk factors in the study groups. ASA score: American Society of Anesthesiologists score; AWD: abdominal wound dehiscence; non-AWD: non-abdominal wound dehiscence

Risk factor	AWD group (n = 59)	Non-AWD group (n = 92)	Test statistics	p-value
	Frequency (%)			
Hemoglobin (<10 g/dL)	32 (54.2%)	32 (34.8%)	χ² = 4.130	0.044
Serum albumin (<3.5 g/dL)	38 (64.4%)	35 (38.0%)	χ² = 4.718	0.032
White blood cell count (>11×10⁹/L)	26 (44.1%)	22 (23.9%)	χ² = 5.848	0.015
ASA score (≥3)	18 (30.5%)	12 (13.0%)	χ² = 5.427	0.021
Presence of anemia	36 (61.0%)	33 (35.9%)	χ² = 10.390	0.011
Preoperative infection	41 (69.5%)	30 (32.6%)	χ² = 20.693	<0.001
Nutritional supplementation before surgery	19 (32.2%)	15 (16.3%)	χ² = 4.013	0.045

In the study, several surgical factors were significantly different between the AWD and non-AWD groups. The AWD group had a higher proportion of emergency surgeries (83.1%) compared to the non-AWD group (55.4%) (p = 0.017). Midline incisions were more common in the AWD group (81.4%) compared to the non-AWD group (43.5%) (p < 0.001), and small bowel pathology was more frequently observed in the AWD group (54.2%) compared to the non-AWD group (16.3%) (p < 0.001). Surgery duration of over two hours was more prevalent in the AWD group (64.4%) than the non-AWD group (26.1%) (p < 0.001). The AWD group also experienced significantly more intraoperative blood loss (>500 mL) (69.5% vs. 31.5%, p < 0.001) and had a higher use of drains (88.1% vs. 66.3%, p = 0.002). While the use of absorbable sutures was slightly higher in the non-AWD group (70.7% vs. 55.9%, p = 0.051), the type of closure was more commonly layered in the non-AWD group (78.3% vs. 61.0%, p = 0.021). The AWD group had more contaminated (30.5% vs. 44.6%) and infected wounds (61.0% vs. 34.8%) compared to the non-AWD group, with a significantly lower rate of clean wounds (8.5% vs. 20.7%, p < 0.001). Stoma formation was significantly higher in the AWD group (25.4% vs. 0.0%, p = 0.001), and the RRI score was significantly higher in the AWD group (4.51 ± 0.86 vs. 2.20 ± 0.79, p < 0.001) (Table 3).

**Table 3:** Intraoperative factors and Rotterdam Risk Index scores. AWD: abdominal wound dehiscence; non-AWD: non-abdominal wound dehiscence

Variable	AWD group (n = 59)	Non-AWD group (n = 92)	Test statistics	p-value
	Frequency (%)/mean ± SD			
Type of surgery				
Elective	10 (16.9%)	41 (44.6%)	χ² = 7.476	0.017
Emergency	49 (83.1%)	51 (55.4%)		
Type of incision				
Midline	48 (81.4%)	40 (43.5%)	χ² = 17.517	<0.001
Others	11 (18.6%)	52 (56.5%)		
Surgery				
Small bowel pathology	32 (54.2%)	15 (16.3%)	χ² = 15.116	<0.001
Others	27 (45.8%)	77 (83.7%)		
Surgery duration (>2 hours)	38 (64.4%)	24 (26.1%)	χ² = 19.365	<0.001
Intraoperative blood loss (>500 mL)	41 (69.5%)	29 (31.5%)	χ² = 21.634	<0.001
Use of drains	52 (88.1%)	61 (66.3%)	χ² = 9.503	0.002
Suture material used				
Absorbable	33 (55.9%)	65 (70.7%)	χ² = 3.723	0.051
Non-absorbable	26 (44.1%)	27 (29.3%)		
Type of closure				
Layered	36 (61.0%)	72 (78.3%)	χ² = 5.301	0.021
Single	23 (39.0%)	20 (21.7%)		
Wound classification				
Clean	5 (8.5%)	19 (20.7%)	χ² = 8.535	<0.001
Contaminated	18 (30.5%)	41 (44.6%)		
Infected	36 (61.0%)	32 (34.8%)		
Stoma formation	15 (25.4%)	0 (0.0%)	χ² = 15.466	0.001
Rotterdam Risk Index score	4.51 ± 0.86	2.20 ± 0.79	t = 8.654	<0.001

Postoperative outcomes showed significant differences between the AWD and non-AWD groups. The incidence of SSIs was notably higher in the AWD group (25.4%) compared to the non-AWD group (3.3%) (p = 0.001). The AWD group also had a higher incidence of seromas (16.9% vs. 5.4%, p = 0.029), while the presence of hematomas was similar between the groups (13.6% vs. 7.6%, p = 0.239). Re-exploration surgery was more common in the AWD group (6.8% vs. 2.2%, p = 0.161), although not significantly. The use of negative-pressure wound therapy (NPWT) was more frequent in the AWD group (10.2% vs. 2.2%), but the difference was not statistically significant (p = 0.075). Postoperative albumin levels were lower in the AWD group (2.78 ± 0.54 vs. 3.05 ± 0.45, p = 0.013). Mechanical ventilation was more commonly required in the AWD group (20.3% vs. 3.3%, p < 0.001), and the postoperative day of dehiscence occurred earlier in the AWD group (5.1 ± 1.7 vs. 6.3 ± 2.1, p = 0.048). Wound discharge by postoperative day 3 was significantly higher in the AWD group (32.2% vs. 13.0%, p = 0.027). Postoperative cough was less frequent in the AWD group (3.4% vs. 5.4%, p = 0.607) (Table 4).

**Table 4:** Postoperative factors and outcomes. AWD: abdominal wound dehiscence; non-AWD: non-abdominal wound dehiscence

Postoperative variable	AWD group (n = 59)	Non-AWD group (n = 92)	Test statistics	p-value
	Frequency (%)			
Development of surgical site infection (SSI)	15 (25.4%)	3 (3.3%)	χ² = 15.373	0.001
Presence of seroma	10 (16.9%)	5 (5.4%)	χ² = 4.802	0.029
Presence of hematoma	8 (13.6%)	7 (7.6%)	χ² = 1.385	0.239
Need for re-exploration surgery	4 (6.8%)	2 (2.2%)	χ² = 2.103	0.161
Use of negative-pressure wound therapy (NPWT)	6 (10.2%)	2 (2.2%)	χ² = 3.244	0.075
Postoperative albumin levels (g/dL)	2.78 ± 0.54	3.05 ± 0.45	t = -2.511	0.013
Postoperative mechanical ventilation	12 (20.3%)	3 (3.3%)	χ² = 15.419	<0.001
Postoperative day of dehiscence	5.1 ± 1.7	6.3 ± 2.1	t = -2.017	0.048
Wound discharge by post-op day 3	19 (32.2%)	12 (13.0%)	χ² = 7.772	0.027
Postoperative cough	2 (3.4%)	5 (5.4%)	χ² = 0.274	0.607

The diagnostic performance metrics for the RRI in predicting adverse wound outcomes (AWDs) are as follows: sensitivity was 100%, indicating perfect detection of AWD cases, while specificity was 90.2%, reflecting a high ability to correctly identify non-AWD cases. The positive predictive value (PPV) was 94.1%, meaning that a high proportion of patients with a positive RRI result actually experienced AWD. The negative predictive value (NPV) was also 100%, indicating that all patients with a negative RRI result did not develop AWD. The area under the ROC curve (AUC) was 0.986, demonstrating excellent overall accuracy, and Youden’s index was 0.902, indicating a strong balance between sensitivity and specificity. The best cut-off for RRI was ≥3.2 (Table 5).

**Table 5:** Predictive accuracy of the Rotterdam Risk Index model.

Metric	Value
Sensitivity (%)	100
Specificity (%)	90.2 (87.5-92.9%)
Positive predictive value (PPV) (%)	94.1 (91.2-96.3%)
Negative predictive value (NPV) (%)	100
Area under the ROC curve (AUC)	0.986
Youden’s index	0.902
Best cut-off for RRI	≥3.2

The multivariate logistic regression analysis revealed several factors significantly associated with AWDs. Serum albumin levels <3.5 g/dL (OR: 1.9, 95% CI: 1.1-3.3, p = 0.021) and hemoglobin levels <10 g/dL (OR: 2.5, 95% CI: 1.2-5.0, p = 0.044) were associated with an increased risk of AWDs. Emergency surgery was a strong predictor (OR: 4.1, 95% CI: 1.5-10.4, p = 0.017). An RRI score >3.2 was the most significant risk factor (OR: 5.1, 95% CI: 3.0-8.3, p < 0.001). The development of SSIs was strongly correlated with AWDs (OR: 6.2, 95% CI: 3.5-10.9, p < 0.001). Longer surgery duration (>2 hours, OR: 2.8, 95% CI: 1.6-4.9, p = 0.002) and intraoperative blood loss >500 mL (OR: 3.3, 95% CI: 1.8-6.1, p < 0.001) were also identified as significant risk factors for AWD (Table 6).

**Table 6:** Logistic regression analysis of risk factors for abdominal wound dehiscence. SSI: surgical site infection

Variable	Odds ratio (OR)	95% confidence interval (CI)	p-value
Serum albumin <3.5 g/dL	1.9	1.1–3.3	0.021
Hemoglobin <10 g/dL	2.5	1.2–5.0	0.044
Emergency surgery	4.1	1.5–10.4	0.017
Rotterdam Risk Index score >3.2	5.1	3.0–8.3	<0.001
Development of SSI	6.2	3.5–10.9	<0.001
Duration of surgery >2 hours	2.8	1.6–4.9	0.002
Intraoperative blood loss >500 mL	3.3	1.8–6.1	<0.001

## Discussion

WD remains a major complication after surgery, especially following exploratory laparotomy. This study aimed to identify risk factors for WD and assess the predictive accuracy of the Rotterdam risk score. We found that age, gender, wound contamination, type of surgery, and comorbidities were significant risk factors. Furthermore, the Rotterdam risk score showed high sensitivity and specificity in predicting WD preoperatively.

Interestingly, the highest incidence of WD was observed in patients under 40 years of age, accounting for 54.2% of cases. This finding is surprising, as older age is traditionally considered a risk factor for WD. However, this trend may be attributed to the higher prevalence of acute abdominal conditions in younger patients, which could lead to increased surgical interventions and subsequent complications. Studies by Yadav et al. and Mahey et al. support this observation, reporting WD rates of 52% and 48%, respectively, among patients younger than 40 years presenting with acute conditions [12,13]. These findings highlight a potential link between acute abdominal conditions and WD in younger populations, possibly compounded by regional healthcare patterns, such as delayed presentation or limited access to early surgical care. Male gender also emerged as a significant risk factor, likely due to factors like smoking and increased abdominal wall tension, in line with studies by Lozada Hernández et al. and Parsa et al. [14,15].

COPD and smoking were significant contributors to WD, impairing collagen synthesis and oxygenation, as confirmed by Fan et al. and Murugavel et al. [16,17]. Low serum albumin levels (<3.5 g/dL) were associated with increased risk (OR: 1.9, 95% CI: 1.1-3.3, p = 0.021), aligning with Naga Rohith et al., who identified hypoalbuminemia as a risk factor for surgical complications [18]. Similarly, anemia (hemoglobin <10 g/dL) doubled the odds of WD (OR: 2.5, 95% CI: 1.2-5.0, p = 0.044), as anemia compromises oxygen delivery and immune function, a finding supported by Ramanachalam et al. [19].

Emergency surgery was another significant factor, increasing the risk of WD (OR: 4.1, 95% CI: 1.5-10.4, p = 0.017). Studies by Walming et al. and Modi et al. found that emergency surgeries often lead to poorer outcomes due to their urgency and complexity [20,21].

Intraoperative factors such as surgery duration (>2 hours) and blood loss (>500 mL) were strongly associated with WD. Prolonged surgeries and significant blood loss can lead to systemic inflammatory responses, hindering wound healing, as demonstrated by Ylimartimo et al. [22]. In addition, small bowel surgeries had a higher incidence of WD, consistent with findings from Sakari et al. and Verma et al., who noted higher complication rates in small bowel surgeries, especially when associated with contamination [23,24].

Wound contamination was a key factor, with 74.5% of cases showing dirty wounds, supporting Fiore et al., who found that contaminated wounds are linked to higher SSI rates and WD [25]. The RRI, which includes age, ASA score, and comorbidities, was a strong predictor of WD (OR: 5.1, 95% CI: 3.0-8.3, p < 0.001), similar to findings by Gonzalez et al., validating the RRI's utility in predicting WD in abdominal surgeries [26].

Postoperative complications were more common in the WD group. SSI occurred in 25.4% of WD patients, compared to 3.3% in non-WD patients (p = 0.001). Infection-induced inflammation leads to collagen degradation, as Vasalou et al. explained, impairing wound healing. The incidence of seromas and wound discharge was also higher in the WD group, indicating fluid accumulation and delayed healing in at-risk patients [27]. Mechanical ventilation was needed for 20.3% of WD patients, compared to 3.3% in the non-WD group (p < 0.001), which is consistent with findings by Arya et al., who identified a higher likelihood of respiratory complications in high-risk patients [28].

The use of drains (88.1% in WD patients) did not significantly affect the rate of SSIs, suggesting that while drains are common, they may not be directly linked to improved wound healing. Similarly, the use of NPWT trended higher in the WD group but did not show a statistically significant difference, warranting further exploration. 

SSIs are among the most frequent and debilitating complications following colorectal surgery, significantly impacting patient recovery and healthcare systems. The prevalence of SSIs in colorectal surgery has been extensively documented, with rates ranging between 10% and 30% in various studies, depending on the population and healthcare settings [29]. SSIs contribute to increased morbidity, prolonged hospital stays, readmissions, and higher healthcare costs, along with the risk of severe complications such as sepsis and, in rare cases, death. In addition, SSIs impose a significant psychological burden on patients, causing distress and impeding their recovery. This underscores the importance of early identification and management strategies to mitigate their occurrence. These findings are consistent with a recent study by Mulita et al., highlighting the widespread burden of SSIs in colorectal procedures [29].

The RRI demonstrated excellent predictive value in our study, with 100% sensitivity, 90.2% specificity, and an area under the ROC curve of 0.986, reaffirming its utility for preoperative risk assessment. Its ease of application, reliance on readily available clinical parameters, and cost-effectiveness make it particularly advantageous in resource-constrained settings. As highlighted by Gómez Díaz et al. and Aggarwal et al., the RRI enables efficient risk stratification, allowing clinicians to prioritize interventions for high-risk patients even in facilities with limited resources [30,31]. This simplicity and cost-efficiency are crucial for enhancing surgical outcomes in underserved regions.

Emerging evidence has highlighted the utility of novel biomarkers in predicting postoperative complications in colorectal surgery. One such promising marker is butyrylcholinesterase (BuChE), an enzyme known for its role in inflammatory pathways and nutritional status. A recent study by Verras et al. suggests that reduced BuChE levels may serve as a predictive indicator of adverse postoperative outcomes, including SSIs and delayed wound healing [32]. Incorporating BuChE level monitoring into preoperative evaluations could aid in identifying high-risk patients, thereby enabling tailored perioperative interventions.

Clinical implications

In our study, 22 patients who developed WD were treated with resuturing, while two required reoperation for anastomotic leaks. The remaining patients were managed conservatively, with healing occurring by secondary intention. Three patients developed entero-atmospheric fistulas, which were also treated conservatively. Despite these complications, no fatalities occurred during the study period, underscoring the importance of appropriate management strategies. Early identification of high-risk patients, particularly those with low serum albumin, anemia, and those undergoing emergency surgeries, facilitates the implementation of tailored perioperative interventions. Proven strategies such as preoperative nutritional optimization, anemia correction using iron supplementation or erythropoiesis-stimulating agents, and perioperative carbohydrate loading have shown efficacy in mitigating surgical risks. Moreover, intraoperative measures like minimizing blood loss and reducing surgery duration through meticulous technique and advanced hemostatic tools can further improve outcomes. The integration of the RRI into preoperative protocols enables the identification of patients who may benefit from enhanced recovery protocols, including multimodal analgesia, early mobilization, and aggressive management of postoperative complications, thus contributing to better overall patient care.

Limitations and future research

While the study provides valuable insights into the predictive utility of the RRI, several limitations must be acknowledged. The retrospective nature of the study may introduce selection bias, potentially affecting the performance metrics of the RRI. For instance, the observed high sensitivity and specificity might be influenced by the characteristics of the selected patient cohort, which may not be representative of all surgical populations. To address these concerns, prospective validation studies in diverse and larger populations are essential to confirm the RRI's generalizability and reliability in varied clinical settings. Moreover, targeted interventions for high-risk patients warrant further investigation. NPWT and optimal drain management are promising approaches to reduce postoperative complications, but robust evidence from randomized controlled trials (RCTs) is necessary to establish their effectiveness across diverse surgical populations. Conducting RCTs would not only strengthen the evidence base but also facilitate the development of standardized protocols for managing high-risk surgical patients, thereby improving outcomes and resource utilization in clinical practice.

## Conclusions

This study underscores the importance of identifying and addressing risk factors for WD, particularly in emergency surgical settings. The RRI proved to be a reliable tool for preoperatively assessing the risk of WD, enabling early intervention and improved patient outcomes. Proactive measures to manage modifiable risk factors, such as correcting anemia, implementing robust infection control measures, and optimizing nutritional status, should be integrated into routine preoperative workflows to reduce the incidence of this debilitating complication. Implementing the RRI in standard preoperative assessments can help clinicians systematically address risk factors and tailor interventions for high-risk patients. Interestingly, the study revealed unexpected demographic insights, with the highest WD rates observed among patients under 40 years of age. This finding challenges traditional assumptions that older age is a primary risk factor and suggests that younger patients may have unique vulnerabilities, potentially linked to the higher prevalence of acute conditions or delays in seeking care in certain healthcare settings. These demographic trends highlight the need for further research to refine risk stratification models and adapt clinical priorities accordingly. Future research should focus on multicenter, prospective studies to validate the RRI’s utility across diverse surgical populations, including low-resource settings, where its ease of application and cost-effectiveness could provide significant clinical advantages. In addition, evaluating refinements to the RRI and incorporating it into comprehensive perioperative care protocols could further enhance its impact. Testing interventions such as NPWT and targeted nutritional strategies in RCTs will also be critical in developing evidence-based approaches to mitigate postoperative risks and improve surgical outcomes globally.
